# Supplementary material for: Functional Characterization of a Phf8 Processed Pseudogene in the Mouse Genome
Source: Genes (Basel). 2023 Jan 8;14(1):172. doi: 10.3390/genes14010172 (PMC9859284; doi:10.3390/genes14010172)
Supplement: Supplementary file 1 [file genes-14-00172-s001.zip › genes-2067685-supplementary.pdf]

Supplemental Material Legends

Supplemental Figure S1. Subcellular localization of PHF8-PS.

Co-immunostaining images of NIH3T3 cells transiently expressing HA/FLAG-tagged PHF8 and PHF8-PS showing the subcellular localization of these proteins (green) and that of the endoplasmic reticulum markers P4HB **(A)** and Calnexin **(B)** (red). Cell nuclei were stained DAPI (blue). Scale bar: 10 μm.

Supplemental Figure S2. Original immunoblotting images.

Original immunoblotting images used for generating Figure 2D **(A)**, Figure 3 **(B-C)** and Figure 4B **(D)**. Red frames indicate the image sections selected for each figure.

Supplemental Table S1. PHF8 and PHF8-ps interacting proteins.

Supplemental Figure S1

A

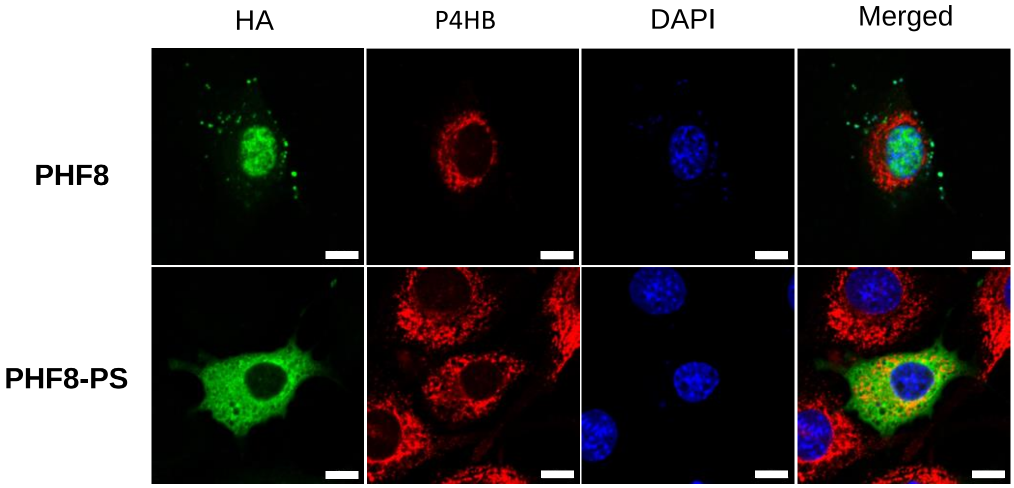

B

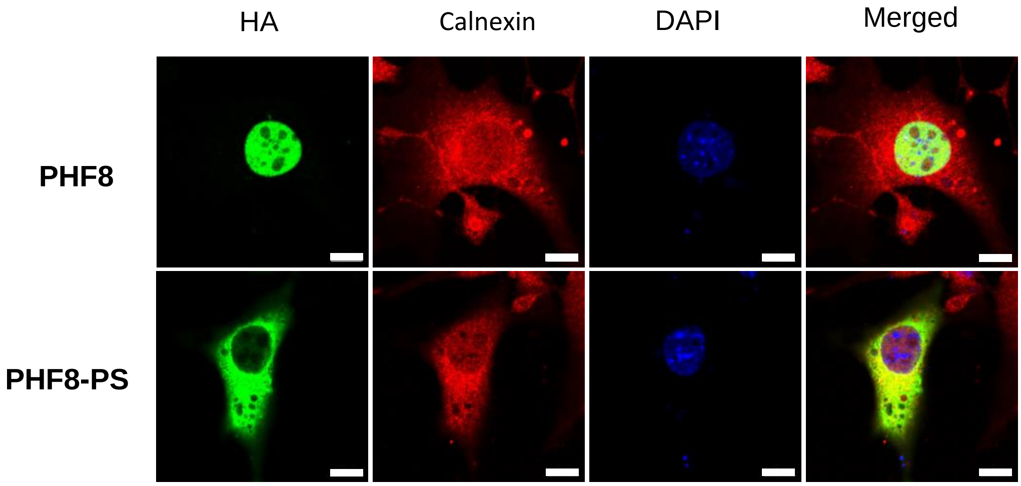

Supplemental Figure S2

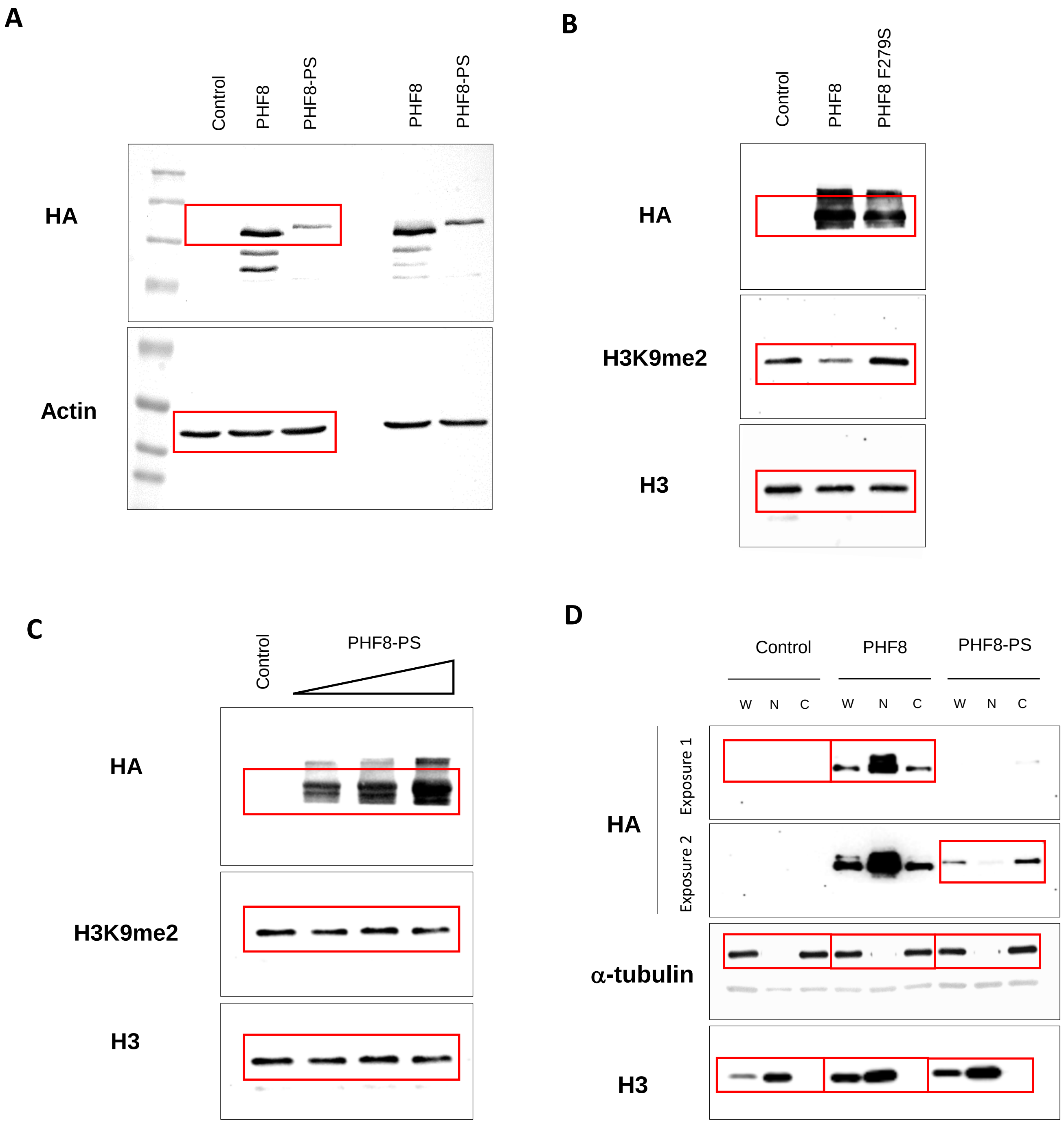

Supplemental Table S1

PHF8-specific interacting proteins	PHF8-ps-specific interacting proteins	PHF8 and PHF8-PS common interacting proteins
Smc2	Atp1a3	Polr2a
Tdrkh	Rhot2	Bag1
Tmem165	Pdpk1	Abcf2
Atp5h	Scfd1	Ntpcr
Smc4	Gfpt1	Dnaja3
Asun	Cul2	Yme1l1
Smc1a	Fkbp10	Cbr4
Psme4	Ndufa10	Faf2
Edc3	Qki	Gcn1l1
Sin3a	Abce1	Slc27a4
Iqsec1	Ears2	Phkb
Far1	Pfdn6	Sucla2
Arcn1	Pcna	Tubg1
Ints10	Stub1	Slc25a12
Hk2	P4hb	Emd
Obfc1	Pdcl2	Bag6
Ncapd3	Tubb4a	Ubxn1
Kif27	Vbp1	Sec61a1
Tecr	Btaf1	Copb1
Tusc3	Fam78a	Fyn
Rad50	Hars	Araf
Mcm5	Pfdn2	Slc25a22
Kifap3	Irak1	Ythdf2
Psma7	Nsun2	Tmem33
Nup188	Ambra1	Polr2c
Rrp12	Stip1	Aifm1
Fanci	Aldh18a1	Sec22b
Psmb2	Cnp	Dnajc11
Nf1	Ipo5	Prkdc
Irs4	Elp3	Clpb
Pomgnt2	Phkg2	Wwox
Ccdc47	Praf2	Xpo1
Stt3a	Sdf4	Gnai3

Acad9	Mad2l1	Ass1
Atp6v1d	Ipo7	Dpm1
Psmb1	Fem1b	Dnajb12
Nsf	Maip1	Ndufs2
Hyou1	Pex1	Afg3l2
Dhcr7	Nampt	Hsph1
Smc3	Ndufs3	Rhot1
Lonp2	Pfdn5	Tubb6
Hs2st1	Galk1	Slc25a11
Gtf2b	Mlf2	Vps4a
Topbp1	Dnaaf2	Ints6
Mark2	Akap8l	Mcm7
Copb2		Arl1
Cby1		Cacybp
Mogs		Gnb1
Ctc1		Tomm22
Cox15		Phgdh
Atp2b1		Slc25a13
Timm50		Ppp6c
Fbxw11		Lrrc41
Pnpla6		Cdipt
Simc1		Fkbp8
Csnk1g3		Pfkl
Bcs1l		Cse1l
Chchd3		Maged2
Abhd12		Huwe1
Pih1d1		Glud1
Fam91a1		Pla2g6
Sacm1l		Polr2b
Xrn2		Copg2
Pigt		Surf4
Ap3m1		Raf1
Tubgcp4		Ahsa1
Sgpl1		Kdelr2
Srpr		Wdr11
Smc6		
Wdr48		
Ncln		
